# Bacterial communities in surface and basal ice of a glacier terminus in the headwaters of Yangtze River on the Qinghai–Tibet Plateau

**DOI:** 10.1186/s40793-022-00408-2

**Published:** 2022-03-26

**Authors:** Ze Ren, Hongkai Gao, Wei Luo, James J. Elser

**Affiliations:** 1 Advanced Institute of Natural Sciences, Beijing Normal University, Zhuhai, 519087 China; 2 School of Environment, Beijing Normal University, Beijing, 100875 China; 3 Key Laboratory of Geographic Information Science (Ministry of Education), East China Normal University, Shanghai, 200241 China; 4 School of Geographic Sciences, East China Normal University, Shanghai, 200241 China; 5 Polar Research Institute of China, Ministry of Natural Resources, Shanghai, 200136 China; 6 School of Oceanography, Shanghai Jiao Tong University, Shanghai, 200030 China; 7 Flathead Lake Biological Station, University of Montana, Polson, 59860 USA

**Keywords:** Glacier terminus, Qinghai–Tibet Plateau, 16S rRNA, Biogeochemical cycles, Nutrient

## Abstract

**Background:**

On the front lines of climate change, glacier termini play crucial roles in linking glaciers and downstream ecosystems during glacier retreat. However, we lack a clear understanding of biological processes that occur in surface and basal ice at glacier termini.

**Methods:**

Here, we studied the bacterial communities in surface ice and basal ice (the bottom layer) of a glacier terminus in the Yangtze River Source, Qinghai–Tibet Plateau.

**Results:**

Surface and basal ice harbored distinct bacterial communities but shared some core taxa. Surface ice communities had a higher α-diversity than those in basal ice and were dominated by Proteobacteria, Firmicutes, Bacteroidetes, Actinobacteria, and Cyanobacteria while basal ice was dominated by Firmicutes and Proteobacteria. The bacterial communities were also substantially different in functional potential. Genes associated with functional categories of cellular processes and metabolism were significantly enriched in surface ice, while genes connected to environmental information processing were enriched in basal ice. In terms of biogeochemical cycles of carbon, nitrogen, phosphorus, and sulfur, bacterial communities in surface ice were enriched for genes connected to aerobic carbon fixation, aerobic respiration, denitrification, nitrogen assimilation, nitrogen mineralization, sulfur mineralization, alkaline phosphatase, and polyphosphate kinase. In contrast, bacterial communities in basal ice were enriched for genes involved in anaerobic carbon fixation, fermentation, nitrate reduction, 2-aminoethylphosphonic acid pathway, G3P transporter, glycerophosphodiester phosphodiesterase, and exopolyphosphatase. Structural equation modeling showed that total nitrogen and environmental carbon:phosphorus were positively while environmental nitrogen:phosphorus was negatively associated with taxonomic β-diversity which itself was strongly associated with functional β-diversity of bacterial communities.

**Conclusions:**

This study furthers our understanding of biogeochemical cycling of the mountain cryosphere by revealing the genetic potential of the bacterial communities in surface and basal ice at the glacier terminus, providing new insights into glacial ecology as well as the influences of glacier retreat on downstream systems.

**Supplementary Information:**

The online version contains supplementary material available at 10.1186/s40793-022-00408-2.

## Introduction

Covering approximately 10% of land surface, glaciers contain 75% of the Earth’s freshwater as ice and are among the least explored environments on Earth [1, 27, 64]. Glacial ecosystems comprise various unique habitats that host diverse and distinctive organisms [8, 58, 62]. As a result of accelerating climate change, glaciers are retreating rapidly [17, 37, 74], losing surface and terminal ice masses as well as associated biotic and abiotic materials to downstream aquatic ecosystems [13, 39, 52, 55]. Given their crucial roles in linking glaciers and downstream ecosystems and fleeting fate during glacier retreat, glacial termini require better study, especially from the perspectives of the microbial communities and their functional potentials in biogeochemical processes.

In glacier termini, surface ice and basal ice exhibit distinct physicochemical environments [51]. The surface of the glaciers is available habitat for primary producers and associated heterotrophic bacterial communities [4, 62]. Glacier algae act as the dominant primary producers to generate autochthonous organic carbon in the upper ice layer [71]. The local heterotrophic bacterial communities as well as downstream biota are fueled by this carbon, playing important biogeochemical roles [4, 52]. Despite the absence of sunlight, subglacial environments, the basal portion of glaciers, also harbor unique but diverse microorganisms [27, 62]. According to previous studies, subglacial environments are dominated by prokaryotic microorganisms, mediating lithotrophic and heterotrophic metabolisms [27, 65]. Due to difficulties in accessing these environments, our knowledge about the taxonomic composition and metabolic potentials of the microbial communities in subglacial environments remains quite limited. Moreover, subglacial and supraglacial environments are directly connected through englacial drainage networks, delivering nutrients, organic matter, and even microorganisms from glacier surface to the base as well as to downstream aquatic ecosystems [19, 39, 50, 52]. However, the differences and connections between the microbial communities of supraglacial and subglacial environments are poorly known.

We conducted the study in a glacier terminus on the Qinghai–Tibet Plateau (QTP). As the “Water Tower of Asia” and the Third Pole, QTP harbors the largest number of glaciers outside polar regions [73]. However, QTP is warming three times faster than the global average in the past 50 years [46]. Thus, the glaciers on the QTP are retreating rapidly and the retreat is accelerating [16, 23, 72]. In this study, we collected surface ice and basal samples from the terminus of Dongkemadi Glacier in the Yangtze River Source Area on the QTP. Our aim is (1) to reveal the taxonomic differences of bacterial communities between surface ice and basal ice, (2) to understand the functional differences in carbon, nitrogen, phosphorus, and sulfur cycling, and (3) to assess the association of nutrient availability (concentrations) and balance (stoichiometric ratios) with taxonomic and functional composition of bacterial communities within the glacier terminus. The melting of terminal ice masses contributes significantly to water sources of glacier-fed aquatic ecosystems, and the differences between surface and basal ice may also influence the composition of biotic and abiotic materials exported to downstream systems. Although this study represents a single glacier, these analyses add to our knowledge of the taxonomic composition and biogeochemical processes of glacial environments influenced by accelerating global climate change.

## Methods

### Study area, field sampling, and chemical analysis

**Fig. 1:**
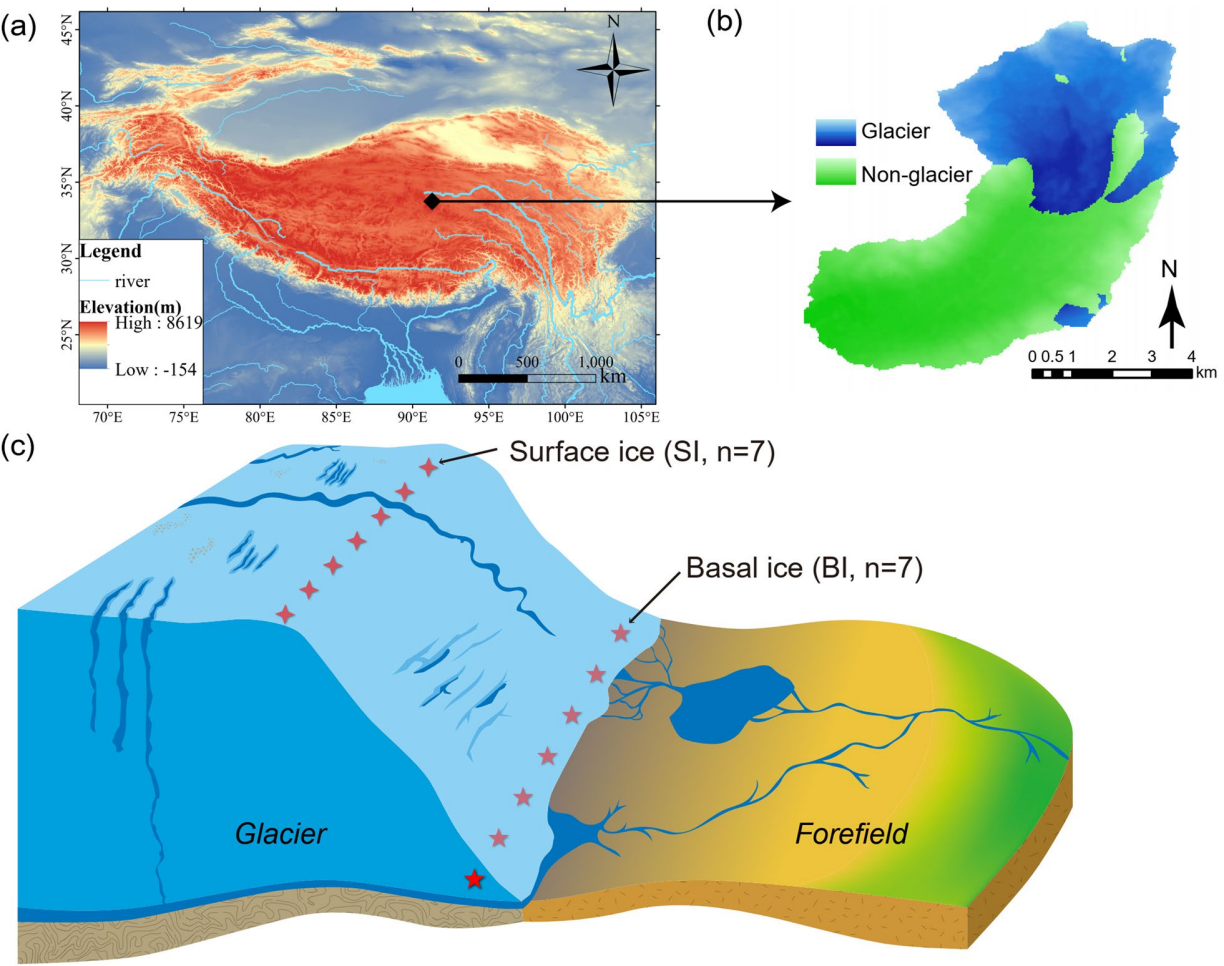
Study area and sampling sites in the glacier terminus of Dongkemadi Glacier. **a** The glacier is located in the Yangtze River headwaters in the central Qinghai–Tibetan Plateau (QTP). **b** Schematic graph showing the glacier area and forefront area of the Dongkemadi Glacier. **c** Schematic graph shown the sampling of surface ice and basal ice. The map and graphs were modified from our previous publication [51]

Fieldwork was conducted at the terminus of Dongkemadi Glacier (33°04′N, 92°04′E), which is located in the headwaters of the Yangtze River in the central QTP (Fig. 1). Covering an area of 15.98 km^2^, the Donkemadi Glacier has an altitude between 5420 to 5919 m, an annual precipitation of 680 mm, and an annual air temperature of − 8.6 ℃ [10, 15]. Since 1970, Dongkemadi Glacier has retreated rapidly [33]. In early July 2019, surface ice (SI) and basal ice (BI) samples were collected for 7 sites at the terminus of the glacier (Fig. 1). However, for SI and BI, only 6 samples were amplified successfully in the PCR process, respectively. As we described in our early study [51], SI samples were collected at a depth of 0–10 cm on the glacier surface. BI samples were collected 10 cm above the base of the ice sheet. The ice samples were placed in sterile bags, stored in a cooler, and transported to the field station for further processing.

Ice samples were melted under room temperature at the field station. Microbial samples were collected by filtering 200 mL meltwater onto a 0.2-μm polycarbonate membrane filter (Whatman, UK). Filters were frozen in liquid nitrogen immediately and stored at − 80 ℃ in the lab until DNA extraction. pH and conductivity were measured using a multiparameter instrument (YSI ProPlus, Yellow Springs, Ohio). For each sample site, three 1-L acid cleaned bottles were filled with meltwater and transported to the laboratory for chemical analyses. Dissolved organic carbon (DOC) was analyzed using filtered (using pre-combusted GF/F filters) meltwater on a Shimadzu TOC Analyzer (TOC-VCPH, Shimadzu Scientific Instruments, Columbia, Maryland). Total nitrogen (TN) and total phosphorus (TP) were measured using the unfiltered water. TN was measured using ion chromatography after persulfate oxidation. TP was measured by using the ascorbate acid colorimetric method after oxidation. The details of these methods and findings are summarized elsewhere [51].

### DNA extraction, PCR, and sequencing

DNA from the filter samples was extracted using the DNeasy PowerSoil Kit (QIAGEN, Germany) following the manufacturer’s protocols. Meanwhile, three blank controls were conducted using the same processes for the samples in order to eliminate the laboratory contamination. The forward primer 343F 5′-TACGGRAGGCAGCAG-3′ and reverse primer 798R 5′-AGGGTATCTAATCCT-3′ [43] were used to amplify the hypervariable V3-V4 regions of bacterial 16S rRNA genes. The PCR amplifications were performed using the following procedure: 5 min initial denaturation at 94 °C, 24 cycles of denaturation at 94 °C for 30 s followed by 30 s annealing at 56 °C and 20 s extension at 72 °C, and a final extension for 5 min at 72 °C. PCR products were verified using agarose gel electrophoresis, purified using the AMPure XP beads (Beckman, USA), and quantified using Qubit dsDNA assay kit (Thermo Fisher Scientific, USA). The amplicon libraries were sequenced on an Illumina MiSeq platform (Illumina, San Diego, CA, USA) according to manufacturer’s instructions. Raw sequence data can be accessed at the China National Center for Bioinformation (PRJCA005802).

### Sequence processing and functional prediction

Raw sequence data were first preprocessed to detect and cut off ambiguous bases and low-quality sequences (average quality score below 20) using Trimmomatic (version 0.35) [6]. After trimming, FLASH software [53] was used in assembly of paired-end reads according to the following parameters: minimal overlapping of 10 bp, maximum overlapping of 200 bp, and maximum mismatch rate of 20%. Sequences were further de-noised using QIIME 1.9.1 [7] according to the following standards: reads with ambiguous, homologous sequences or below 200 bp were abandoned; reads with 75% of bases above Q20 were retained; reads with chimera were detected and removed. After removing the primer sequences, the clean reads were clustered to generate operational taxonomic units (OTUs) at 97% genetic similarity against the SILVA 132 database [47]. The sequences were rarefied to the same depth of 29,748 sequence per sample to normalize surveying effort using the *single_rarefaction.py* script. The alpha diversity indices, including Chao 1, observed OTUs, Simpson, and Shannon, were calculated using the *alpha_diversity.py* script in QIIME 1.9.1 [7].

The functional profiles of the bacterial communities were predicted from the 16S rRNA gene sequence data using PICRUSt version 1.0 (phylogenetic investigation of communities by reconstruction of unobserved states) [30]. We calculated the nearest sequence taxon index (NSTI), which measures the average phylogenetic distance between OTUs and a gene sequence from a fully sequenced genome [30]. The average NSTI value in this study was 0.082, indicating a high accuracy of the PICRUSt prediction. Although PICRUSt prediction only reflects a potential rather than a realized functional capacity, our data offer an approach to get a glimpse of potential functions of bacterial communities in surface and basal ice. The KEGG orthologs (KOs, https://www.genome.jp/kegg/) were clustered to different pathway levels (levels 1–3) using the predicted metagenomes. The KOs associated with the biogeochemical cycles of carbon, nitrogen, sulfur, and phosphorus were further extracted (Additional file 1: Table S1) and the relative abundance of each pathway was calculated [31, 35].

### Statistical analyses

We compared taxonomic structure and functional potential of bacterial communities in surface ice and basal ice. The phylogenetic tree of all the detected 464 OTUs was built using iTOL (https://itol.embl.de/). Then, non-metric multidimensional scaling was used to reveal the differences of taxonomic and functional composition of bacterial communities between surface ice and basal ice based on the relative abundance of OTUs and KOs, using Vegan package 2.5-7 [44]. Heatmaps were used to illustrate the relative abundances of the core OTUs in both basal ice and surface ice, using pheatmap v.1.0.12 [29]. Structural equation models (SEM) were generated to assess the association of environmental variables with β-diversity of bacterial communities using piecewiseSEM v.2.1.2 [32]. All analyses were conducted in R 3.4.1 [48].

## Results

### Taxonomic diversity and community composition

**Fig. 2:**
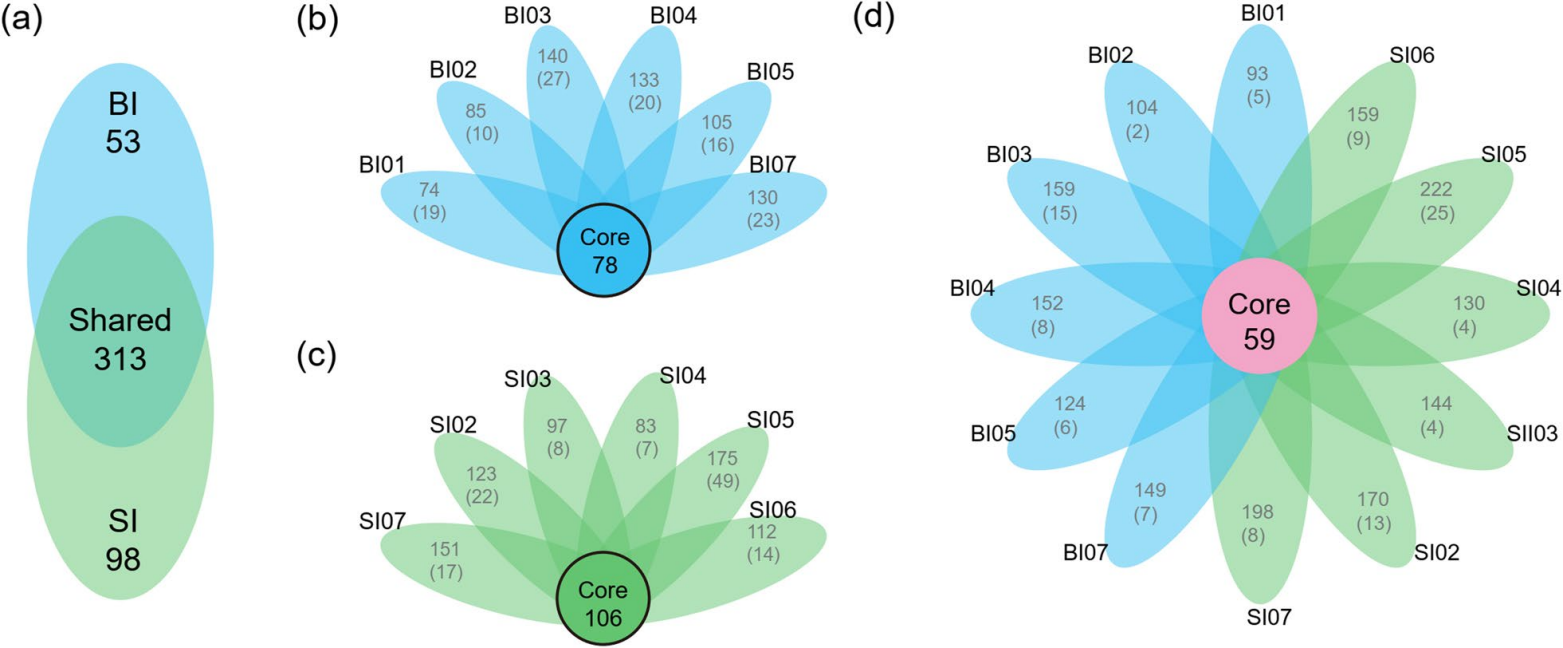
**a** Venn diagram showing the shared and unique OTUs between surface ice and basal ice. **b**–**d** Flower plot diagram showing core and unique OTUs across all samples in basal ice, surface ice, and both. The central circle shows the number of OTUs common to all samples while the petals show the number of OTUs in addition to the core set, as well as the number of OTUs unique to each sample (in brackets)

After quality filtering, 356,976 reads were retained, which were clustered into 464 OTUs at 97% similarity level. Basal ice harbored 366 OTUs, of which, 53 OTUs present only in basal ice samples but not in surface ice samples (Fig. 2a) and 78 were core OTUs presenting in all basal ice samples (Fig. 2b). Surface ice harbored 411 OTUs, of which 98 OTUs were found only in surface ice samples but not in basal ice samples (Fig. 2a). 106 were core OTUs occurring in all surface ice samples (Fig. 2c). Moreover, 311 OTUs were found in both basal and surface ice samples (Fig. 2a), and only 59 OTUs were found in all the samples collected (Fig. 2d).

**Fig. 3:**
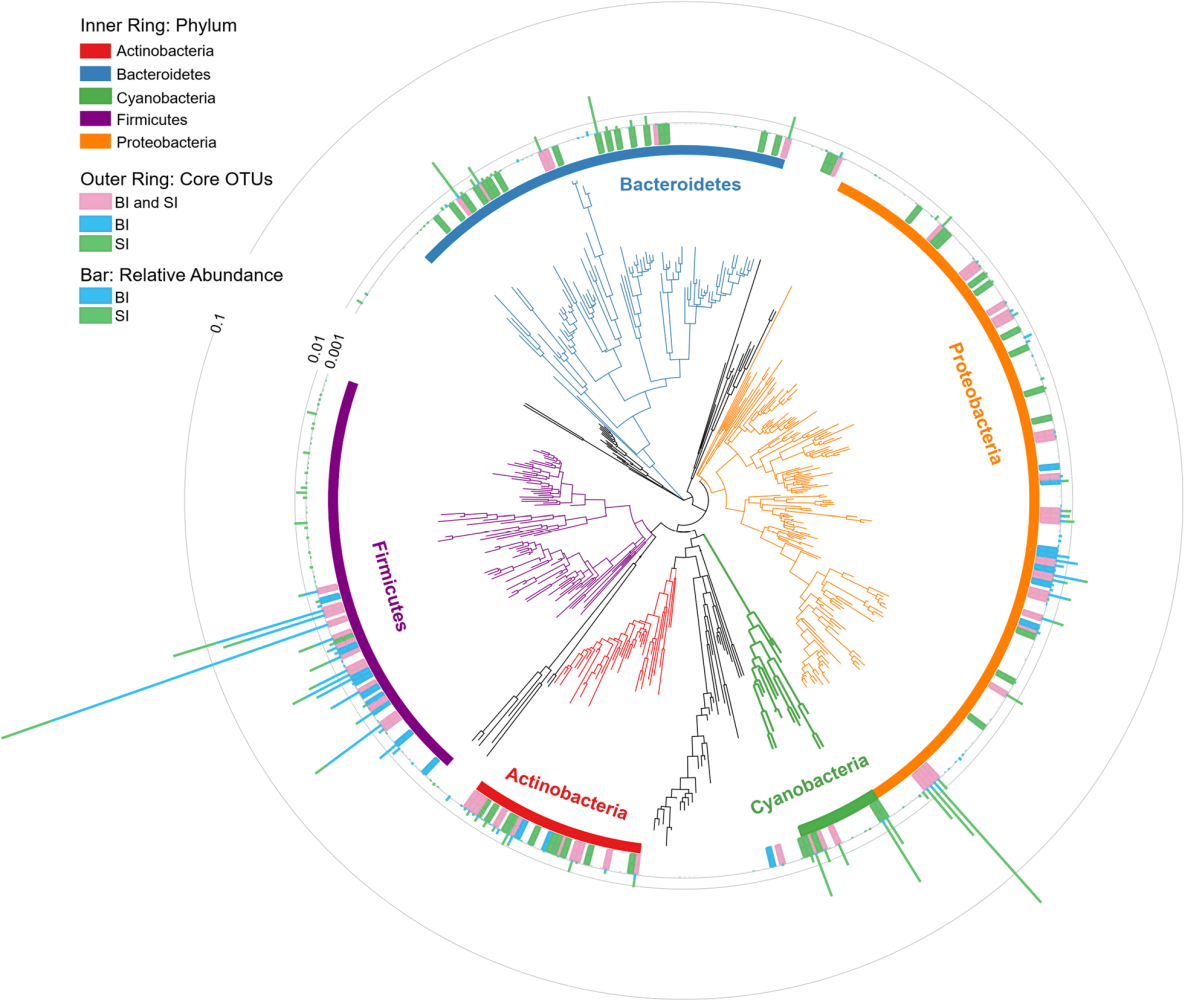
Phylogenetic tree based on 464 OTUs found in basal ice (BI) and surface ice (SI). The tree and the inner ring were colored by taxonomy (phylum). The outer ring denotes the core OTUs found in BI, SI, or both. The bar denotes the relative abundances of the OTUs in BI and SI

Of the 464 OTUs in total, most belonged to five dominant phyla: Proteobacteria (160 OTUs), Firmicutes (90 OTUs), Bacteroidetes (84 OTUs), Actinobacteria (40 OTUs), and Cyanobacteria (18 OTUs) (Fig. 3). The remaining 72 OTUs belonged to 10 other phyla. In surface ice bacterial communities, 24 OTUs had a relative abundance above 1% (Fig. 3 and Additional file 1: Table S2). In basal ice bacterial communities, 19 OTUs had a relative abundance above 1% (Fig. 3 and Additional file 1: Table S2). There were 8 OTUs (7 associated to Firmicutes and 1 associated to Proteobacteria) had a relative abundance above 1% in both surface and basal ice, belonging to the genus *Lactobacillus, Lactococcus, Weissella, Pediococcus, Bacillus, and Polaromonas* (Fig. 3 and Additional file 1: Table S2).

**Fig. 4:**
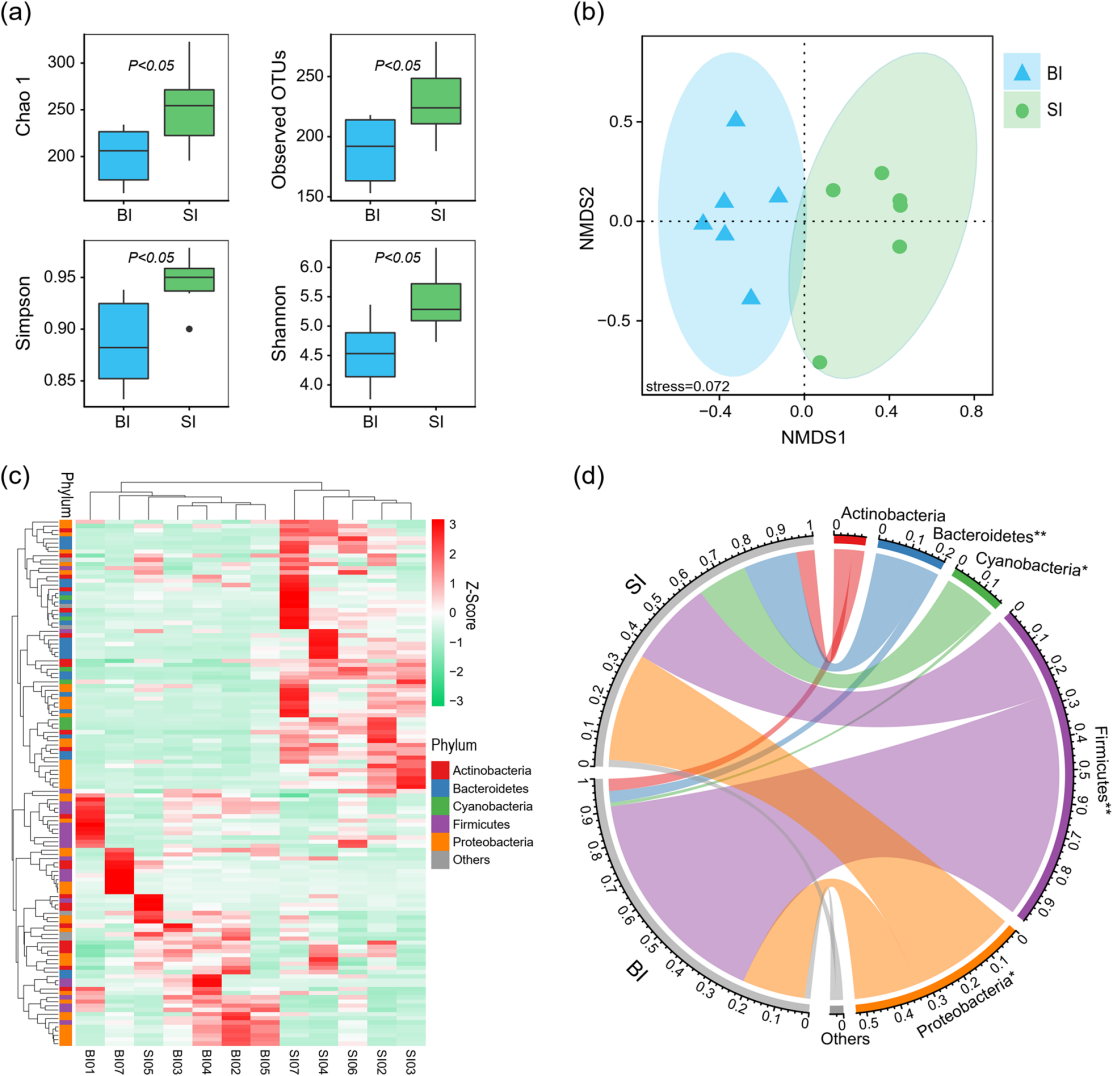
Taxonomic properties of bacterial communities in surface ice (SI) and basal ice (BI). **a** Alpha diversity. **b** Non-metric multidimensional scaling based on the relative abundance of all OTUs. **c** Hierarchical cluster dendrogram for the 59 core OTUs in both basal ice and surface ice based on Bray–Curtis dissimilarity. The colors in the heatmap illustrate the relative abundance of each OTU per sampling site. **d** Bacterial community composition shown at the phylum level

Surface ice had significantly higher alpha diversity than basal ice (Fig. 4a). The average numbers of observed OTUs in surface ice and basal ice were 230 and 189, respectively (Fig. 4a). Surface ice and basal ice had distinct bacterial communities based on NMDS (Fig. 4b). Cluster analysis based on the 59 core OTUs present in all samples confirmed that bacterial community composition was habitat-dependent: surface ice samples fell into one cluster (except SI05) and basal ice samples into another (Fig. 4c).

In surface ice, the most abundant phylum was Proteobacteria (33.6%), followed by Firmicutes (26.7%), Bacteroidetes (16.6%), Cyanobacteria (15.4%), and Actinobacteria (5.6%) (Fig. 4d). In basal ice, however, the most abundant phylum was Firmicutes (70.1%) and Proteobacteria (19.5%) (Fig. 4d). The relative abundance of Proteobacteria, Bacteroidetes, and Cyanobacteria were significantly higher in surface than in basal ice (Fig. 4d). However, Firmicutes was significantly higher in basal ice than in surface ice (Fig. 4d).

### Functional composition

**Fig. 5:**
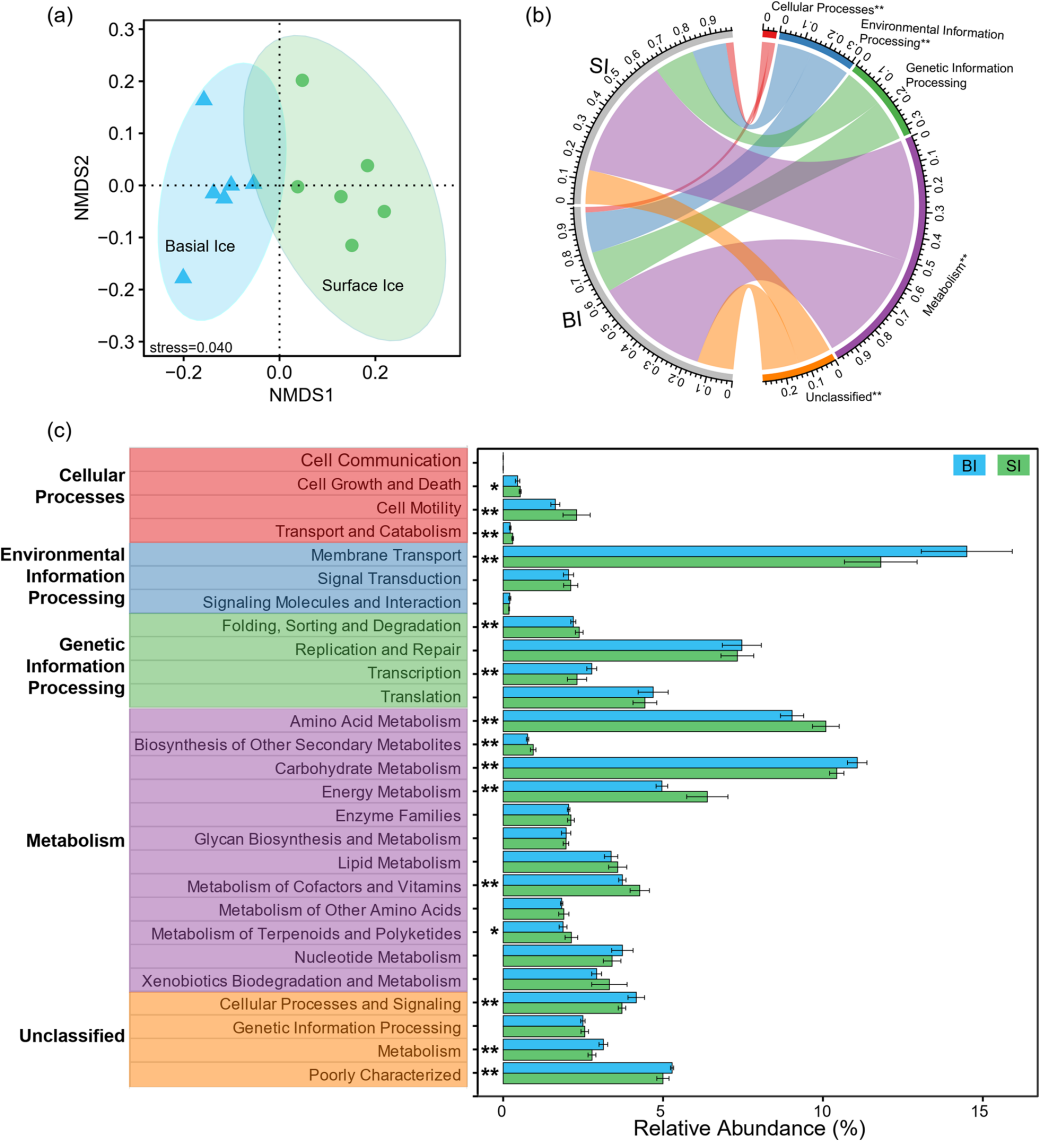
Functional differences between bacterial communities in surface ice and basal ice. **a** Non-metric multidimensional scaling based on the relative abundance of all KOs. **b** KEGG categories at level 1. **c** KEGG categories at level 2. The differences were assessed using Wilcoxon rank-sum test with “*” and “**” represent *p* < 0.05 and *p* < 0.01, respectively

Functional composition of the communities was analyzed according to PICRUSt-predicted KEGG orthologies (KOs). In general, bacterial communities in surface ice and basal ice had significantly different functional compositions (Fig. 5). Notably, at KEGG level 1, cellular processes and metabolism were significantly enriched in surface ice, while environmental information processing was enriched in basal ice (Fig. 5b). At KEGG level 2, bacterial communities in surface ice were significantly enriched for genes associated with cell growth and death, cell motility, transport and catabolism, amino acid metabolism, biosynthesis of other secondary metabolites, energy metabolism, metabolism of cofactors and vitamins, and metabolism of terpenoids and polyketides. Bacterial communities in basal ice were significantly enriched for genes related to membrane transport and carbohydrate metabolism, (Fig. 5c).

### Functional potential in biogeochemical processes for C, N, P, and S

**Fig. 6:**
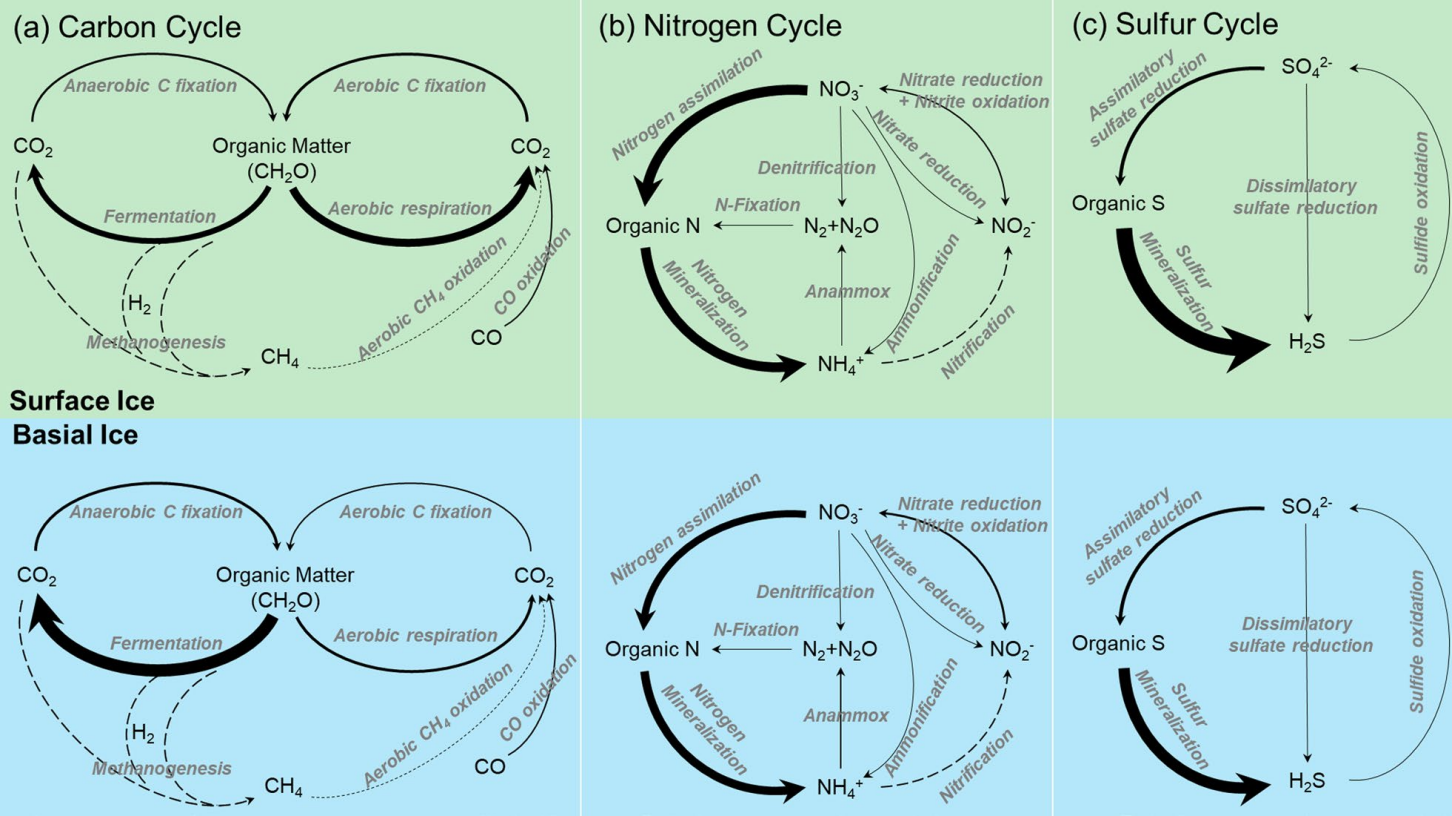
The genetic potential for each conversion step in the **a** carbon, **b** nitrogen, and **c** sulfur cycles in surface ice and basia ice of the glacier terminus was estimated using a combination of normalized marker genes as summarized in Additional file 1: Table S1. The arrow size is proportional to the relative abundance of the KEGG orthologies associated to the pathways. The dotted lines represent the conversion step without marker genes been detected. This figure was modified from [31, 35]

**Fig. 7:**
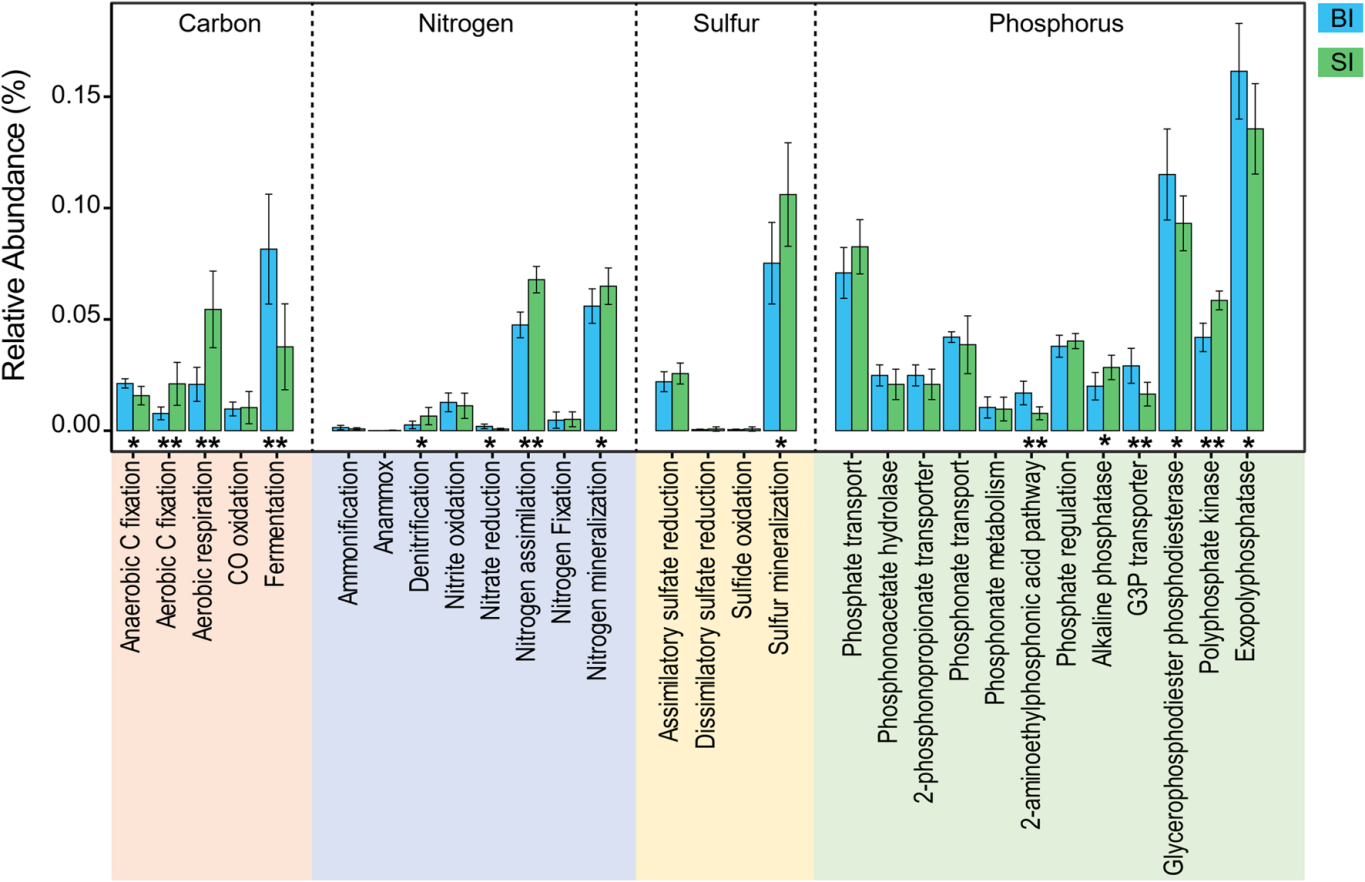
Relative abundance of genes associated with processing of carbon, nitrogen, sulfur, and phosphorus for bacterial communities in surface ice and basal ice. The differences were assessed using Wilcoxon rank-sum test with “*” and “**” represent *p* < 0.05 and *p* < 0.01, respectively

A combination of normalized marker genes (Additional file 1: Table S1) predicted by PICRUSt was used to infer the genetic potential of the conversion steps in biogeochemical cycles of carbon, nitrogen, phosphorus, and sulfur mediated in surface ice and basal ice (Figs. 6, 7). In terms of carbon cycle, bacterial communities in surface ice were enriched with respect to genes for aerobic carbon fixation and aerobic respiration but for anaerobic carbon fixation and fermentation in basal ice (Figs. 6a, 7). For nitrogen cycle, genes involved in denitrification, nitrogen assimilation, and nitrogen mineralization were enriched in surface ice while nitrate reduction genes were enriched in basal ice (Figs. 6b, 7). For sulfur cycle, the bacterial communities in surface and basal ices were only different in genes associated with sulfur mineralization, which was enriched in surface ice (Figs. 6c, 7). In terms of phosphorus cycle, bacterial communities were enriched for genes for alkaline phosphatase and polyphosphate kinase in surface ice but for 2-aminoethylphosphonic acid pathway, G3P transporter, glycerophosphodiester phosphodiesterase, and exopolyphosphatase genes in basal ice (Fig. 7).

### Influences of environmental variables on community variation

**Fig. 8:**
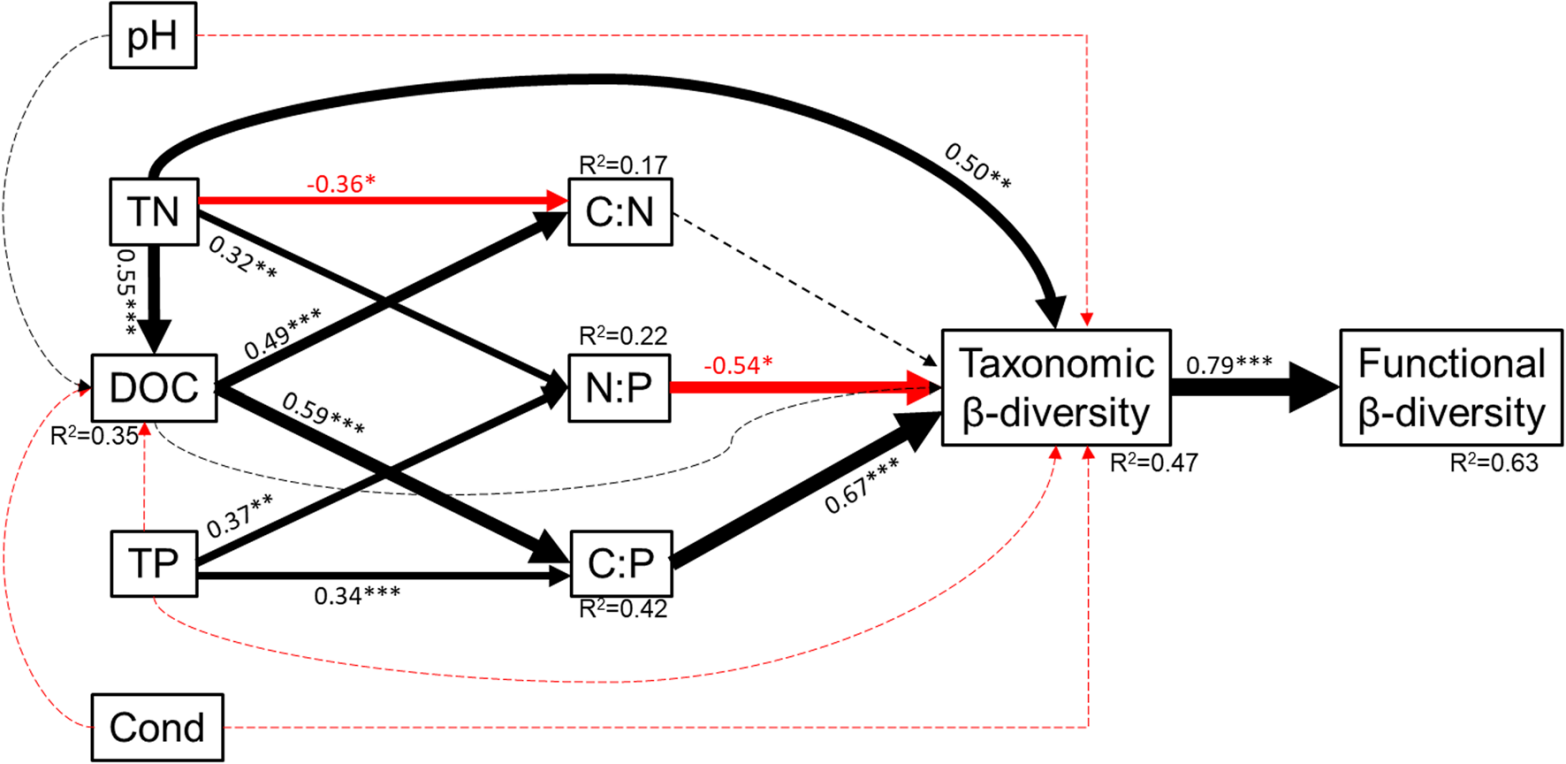
Structural equation model illustrating the direct and indirect associations of nutrient concentrations and stoichiometry on taxonomic β-diversity and functional β-diversity. The solid lines represent significant relationships with red and black arrows represent negative and positive relationships, respectively. Adjacent values near the arrows indicate path coefficients, which were shown proportionally as the width of the arrow. Significance levels are denoted with “*”, “**” and “***” represent *p* < 0.05, *p* < 0.01, and *p* < 0.001, respectively

The results of SEM highlighted direct and indirect influences of environmental variables and nutrient stoichiometry on taxonomic and functional variations (β-diversity) in surface ice and basal ice (Fig. 8). Based on the SEM, only variation in total nitrogen was directly and positively associated with taxonomic β-diversity (Fig. 8). However, TN, DOC, and TP indirectly affected β-diversity via impacts on N:P and C:P ratios, as N:P had a negative relationship while C:P had a positive relationship with taxonomic β-diversity (Fig. 8). Moreover, functional β-diversity was indirectly affected by nutrient and nutrient stoichiometric ratios through their effects on taxonomic β-diversity (Fig. 8).

## Discussion

### Taxonomic properties

In glacier termini, surface ice and basal ice are two distinct environments with substantially different physicochemical properties [51]. Our data show that these habitats also differ in the taxonomic structure and biogeochemical potential of their bacterial communities. Bacterial communities in surface ice had a higher α-diversity than in basal ice and were dominated by Proteobacteria, Firmicutes, Bacteroidetes, Actinobacteria, and Cyanobacteria. Because of exposure to air, surface ice receives abiotic materials [20, 58] as well as microorganisms [5, 21, 42] from atmospheric deposition. The airborne microorganisms develop into distinct communities in supraglacial environments. For photosynthetic biota, cyanobacteria (Leptolyngbyaceae, Phormidiaceae, and Pseudanabaenaceae) were important players (Fig. 3 and Additional file 1: Table S2). Some of these taxa have also been reported on the surface of polar glaciers [25, 63]. In supraglacial environments, cryoconite holes are nutrient-enriched biological hotspots harboring both photosynthetic and various heterotrophic microorganisms [1, 11, 64], that are generally dominated by Proteobacteria, Bacteroidetes, and Actinobacteria [5, 21], consistent with our study.

Bacterial communities in basal ice were dominated by Firmicutes and Proteobacteria, had lower α-diversity, and shared some core OTUs with bacterial communities in surface ice. In contrast with supraglacial environments, basal ice lies in the deepest portion of the glacier with absence of light, lack of energy sources, and even low availability of liquid water [3, 27, 52]. This harsh environment pushes life to its limits, leading to lower bacterial diversity [45, 65]. At the Dongkemadi glacier, the taxa that occurred in basal ice with high relative abundance mostly belonged to Firmicutes and Proteobacteria, including the genera *Bacillus, Lactobacillus, Streptococcus, Lactococcus, Enterococcus,* and *Klebsiella* (Fig. 3 and Additional file 1: Table S2). These bacteria have also been found in other glaciers, indicating their prominent cold adaptability [24, 34, 66] as well as potential anthropogenic pollution, such as antibiotic resistant bacteria, *Enterococcaceae* and *Enterobacteriaceae* [26, 49].

Subglacial ecosystems are associated with supraglacial ecosystems through englacial hydrological networks that transport water, nutrients, and even microbes from the surface to the base of the glacier [14, 19, 22]. Thus, basal ice and surface shared a substantial number of OTUs (Fig. 2a). However, due to major differences in abiotic environments, the basal and surface habitats only shared 59 core OTUs (Fig. 2d). Moreover, basal ice also interacts biotically and abiotically with underlying bedrock/sediments [27, 40, 62], where microbial communities mediate chemical weathering [41, 68]. Thus, basal ice and surface ice had significantly different bacterial communities but shared some taxa.

### Functional potential

In addition to the taxonomic differences, the bacterial communities in surface ice and basal ice also differed in functional composition and relative abundances of genes involved in biogeochemical cycling of C, N, P, and S. Bacterial communities in supraglacial environments had increased representation of genes associated with high metabolic activities, such as genes associated with amino acid metabolism and energy metabolism. In glacial environments, avoiding cell damage caused by ice crystal nucleation is critical [57], with greater importance for bacteria in basal ice than in surface ice. Correspondingly, the KEGG category environmental information processing and especially membrane transport were more enriched in basal ice than in surface ice (Figs. 4, 5), likely due to the need to synthesize cryoprotectants and osmoprotectants [60]. Such differences in functional potential are likely key for survival in these harsh environments, leading to bacterial communities with the distinctive taxonomic composition that we document (Fig. 3).

In surface ice, enrichment of genes involved in aerobic carbon fixation are likely a reflection of high cyanobacteria abundance (Figs. 3, 6). In general, organic carbon is generated by both eukaryote algae and cyanobacteria [5, 62] but only a small proportion of the organic C is utilized by heterotrophic bacteria [4]. Thus, surface ice had significantly higher organic carbon concentrations than basal ice [51]. In terms of functional potential, this dominance of primary production was supported by high relative abundance of Calvin cycle genes for photosynthetic organisms, especially genes encoding phosphoribulokinase (Fig. 6 and Additional file 1: Table S1), an essential photosynthetic enzyme. Due to the oxygenated environment of surface ice, it is expected that aerobic carbon respiration would be enriched in surface ice and mainly dominated by heterotrophic bacteria, such as Proteobacteria and Bacteroidetes (Fig. 6 and Additional file 1: Table S1). In the absence of light, however, bacterial communities in basal ice appear to be supported by lithotrophic and heterotrophic metabolisms [27]. Moreover, because of isolation from the atmosphere, basal ice are generally anoxic environments. Thus, anaerobic metabolisms were prevalent. In our study, for example, bacterial communities in basal ice were enriched for genes involved in anaerobic carbon fixation and fermentation (Figs. 6, 7). Anaerobic carbon fixation would be supported by chemolithotrophy and particulate organic carbon would be decomposed into smaller molecules, ultimately to CO_2_ [9, 27]. Moreover, in both surface ice and basal ice, substantial potential for carbon monoxide (CO) oxidation was found (Figs. 6, 7). In highly oligotrophic environments, CO oxidation is a successful strategy for heterotrophs because CO can serve as both carbon source and electron donor [28]. According to previous studies, most of the bacteria mediating CO oxidation are *Burkholderiaceae* [28, 35, 69], which had high relative abundance in surface ice (Fig. 3 and Additional file 1: Table S1) at Dongkemadi glacier.

In both surface and basal ice, most of the genes involved in the nitrogen cycle appeared to be limited to those involved in assimilation and mineralization (Figs. 6, 7). This is supported by the presence of marker genes encoding glutamine synthetase (GlnA) and glutamate synthases (GltBS) for nitrogen assimilation and marker genes encoding glutamate dehydrogenase (GDH) (Additional file 1: Table S1). Nitrogen assimilation and mineralization pathways were enriched in surface ice, mainly involving diverse heterotrophic bacteria (Betaproteobacteria, Alphaproteobacteria, and Actinobacteria). According to our previous study of the same glacier, ammonium was 4.6 and 6.9 times higher than nitrate in surface and basal ice, respectively [51]. The high level of ammonium could be produced by microbial decomposition of organic matter and might inhibit the expression of nitrogen fixation genes [35]. Marker genes encoding nitrification and denitrification were rare in both environments (Figs. 6, 7). We infer that, because of the absence of nitrification genes, the produced ammonium can be reused by organisms or delivered to downstream environments. In oligotrophic environments, low nitrification can also be a mechanism to conserve bioavailable nitrogen [31].

In glacier environments, phosphorous availability is typically very low [51, 52]. Geochemical analyses of surface ice and basal ice of the Dongkemadi Glacier further revealed very high environmental C:P and N:P ratios and surface ice had even higher environmental C:P and N:P ratios than basal ice, indicating that bacterial communities likely face severe P-limitation, especially in surface ice [51]. Moreover, our previous study showed that soluble reactive phosphorus (SRP) accounted for 38% and 62% of TP in surface and basal ice, respectively [51]. Low TP and SRP concentrations likely help explain enrichment of alkaline phosphatase and polyphosphate kinase genes in surface ice (Figs. 6, 7) as these enzymes allow bacteria to utilize organic phosphorus under phosphorus deficiency [54]. In addition, bacteria have been found to incorporate and process dissolved organic phosphorus (such as phosphonates) in oligotrophic environments [35, 36] via increased expression of phosphonate and G3P (glycerol-3-phosphate) transporters [67]. Furthermore, the genes encoding exopolyphosphatase (a phosphatase enzyme catalyzing the hydrolysis of inorganic polyphosphate), glycerophosphodiester phosphodiesterases (enzymes degrading various glycerophosphodiesters to produce G3P), and G3P transporter were enriched in basal ice, likely to promote organic P utilization.

### Environmental influences

In glacier ecosystems, nutrient supplies are typically poor (especially for P) and imbalanced [2, 51, 52]. Nutrients are the key limiting factors in many ecosystems, and the availability and mass balance of C, N, and P place crucial controls on ecosystem structure and processes [12, 61], especially in the nutrient-deficient cryosphere environment [13, 52]. Constrained nutrient stoichiometry is regarded as one of the general rules of life with strong implications for glacier-influenced lakes and streams [13]. For Dongkemadi glacier, surface ice and basal ice are different in nutrient concentration and C:N:P stoichiometry [51], likely affecting taxonomic and functional properties of bacterial communities (Fig. 8). Indeed, TN and C:P were positively but N:P was negatively associated with taxonomic β-diversity (Fig. 8). While surface ice and basal ice are also different in conductivity and pH [51], bacterial community structure was not correlated with these two environmental variables (Fig. 8), further suggesting the importance of nutrient availability and stoichiometry in structuring bacterial communities in oligotrophic glacier ecosystems. The positive relationships of TN and C:P with β-diversity suggest that these environmental factors drive bacterial community divergence while the negative relationship of β-diversity with N:P suggests that N:P drives convergence of the bacterial communities. This implies that nutrient availability and stoichiometry likely influence the functional potential of bacterial communities by changing their taxonomic composition.

### Implications for downstream systems

Upstream catchments export biotic and abiotic materials from various glacial and non-glacial sources to downstream aquatic ecosystems, substantially influencing ecosystem structure and function of glacier-fed streams and lakes [39, 52, 59, 70]. For example, microorganisms from surface ice and snow, englacial environments, ground water, adjacent soils and rocks, and atmospheric deposition are different and contribute to form the metacommunity of glacier-fed streams [2, 18, 38, 56]. We further revealed that, at the glacier terminus, surface and basal ice had different biotic and abiotic composition as was also indicated in our previous study [51]. The entirely of ice columns at the glacier terminus, from surface to bottom, are melting and exporting water and materials to downstream systems. Thus, meltwater composition derived from different sources (surface ice/snow, englacial ice, basal ice, and ground water, etc.) influences the biotic and abiotic materials exported to downstream systems. However, it is challenging to quantify the composition of these water sources but these potential influences motivate future efforts to do so.

## Supplementary Information

**Additional file 1. **List of the KEGG orthologs associated with carbon, nitrogen, phosphorus, and sulfur cycles. List of OTUs with relative abundance above 1% in surface ice and/or basial ice**.**

**Additional file 2. **OTU table of the sample sites**.**

## Data Availability

Raw sequence data can be accessed at the China National Center for Bioinformation (PRJCA005802).

## Abbreviations

QTP: Qinghai–Tibet Plateau
SI: Surface ice
BI: Basal ice
DOC: Dissolved organic carbon
TN: Total nitrogen
TP: Total phosphorus
OTUs: Operational taxonomic units
KOs: KEGG orthologs
SEM: Structural equation models
