# Supplementary material for: Bacterial communities in surface and basal ice of a glacier terminus in the headwaters of Yangtze River on the Qinghai–Tibet Plateau
Source: Environ Microbiome. 2022 Mar 26;17:12. doi: 10.1186/s40793-022-00408-2 (PMC8962558; doi:10.1186/s40793-022-00408-2)
Supplement: Supplementary file 1 — Additional file 1. List of the KEGG orthologs associated with carbon, nitrogen, phosphorus, and sulfur cycles. List of OTUs with relative abundance above 1% in surface ice and/or basial ice. [file 40793_2022_408_MOESM1_ESM.docx]

**Bacterial communities in surface and basal ice of a glacier terminus in the headwaters of Yangtze River on the Qinghai-Tibet Plateau**

Ze Ren^1,2*^, Hongkai Gao^3,4*^, Wei Luo^5,6^, James J. Elser^7^

1 Advanced Institute of Natural Sciences, Beijing Normal University, Zhuhai, 519087, China

2 School of Environment, Beijing Normal University, Beijing, 100875, China

3 Key Laboratory of Geographic Information Science (Ministry of Education), East China Normal University, Shanghai, 200241, China

4 School of Geographic Sciences, East China Normal University, Shanghai, 200241, China

5 Polar Research Institute of China, Ministry of Natural Resources, Shanghai 200136, China

6 School of Oceanography, Shanghai Jiao Tong University, Shanghai 200030, China

7 Flathead Lake Biological Station, University of Montana, Polson, 59860, USA

***Corresponding Author**:

Ze Ren: [renzedyk@gmail.com](mailto:renzedyk@gmail.com)

Hongkai Gao: [hkgao@geo.ecnu.edu.cn](mailto:hkgao@geo.ecnu.edu.cn)

Table S1 The genetic potential for each conversion step in the carbon, nitrogen, and sulfur cycles in the thermokarst lakes was estimated using a combination of normalized marker genes.

| **KO** | **Gene** |
| --- | --- |
| **Carbon Cycle** | |
| **Anaerobic C fixation (K00174+K00175+K00244+ K01648)/4+(K00194+K00197)/2** | |
| K00174 | 2-oxoglutarate:ferredoxin oxidoreductase subunit alpha |
| K00175 | 2-oxoglutarate:ferredoxin oxidoreductase subunit beta |
| K00244 | frdA; fumarate reductase flavoprotein subunit |
| K01648 | adenosinetriphosphate (ATP) citrate lyase |
| K00194 | CO dehydrogenase subunit delta |
| K00197 | CO dehydrogenase subunit gamma |
| **Aerobic C fixation (Calvin cycle) (K00855+K01602)/2** | |
| K00855 | phosphoribulokinase |
| K01602 | RuBisCO small chain |
| **Aerobic CH4 oxidation** | |
| K08684 | methane monooxygenase |
| **Aerobic respiration (K02256+K02262)/2+(K02274+K02276)/2** | |
| K02256 | cytochrome c oxidase subunit I (coxI) |
| K02262 | cytochrome c oxidase subunit III (coxIII) |
| K02274 | cytochrome c oxidase subunit I (coxA) |
| K02276 | cytochrome c oxidase subunit III (coxC) |
| **CO oxidation (K03518+K03519+K03520)/3** | |
| K03518 | CO dehydrogenase small subunit (coxS) |
| K03519 | cutM, coxM; carbon-monoxide dehydrogenase medium subunit |
| K03520 | cutL, coxL; carbon-monoxide dehydrogenase large subunit |
| **Fermentation K00016 + (K00169 + K00170)/2** | |
| K00016 | L-lactate dehydrogenase |
| K00169 | pyruvate:ferredoxin oxidoreductase alpha subunit |
| K00170 | pyruvate:ferredoxin oxidoreductase beta subunit |
| **Methanogenesis (K00400+K00401)/2** | |
| K00400 | coenzyme M methyl reductase beta subunit (mcrB) |
| K00401 | methyl coenzyme M reductase system, component A2 |
| **Nitrogen Cycle** | |
| **Ammonification K05904+K03385** | |
| K03385 | formate c552 (nrfA) -dependent nitrite reductase periplasmic cytochrome |
| K05904 | cytochrome c nitrite reductase (nrfA) |
| **Anammox (SRAO)** | |
| K10535 | hydroxylamine oxidoreductase/hydrazine oxidoreducatse |
| **Denitrification (K02305+K04561+K00376)/3** | |
| K00376 | nitrous oxide reductase (nosZ) |
| K02305 | nitric-oxide reductase (norC) |
| K04561 | nitric-oxide reductase (norB) |
| **Nitrate reduction + Nitrite oxidation (K00370+K00371)/2** | |
| K00370 | nitrate reductase alpha & nitrite oxidoreductase (narG/nxrA) |
| K00371 | nitrate reductase beta & nitrite oxidoreductase (narH/nxrB) |
| **Nitrate reduction (K02567+K02568)/2** | |
| K02567 | periplasmic nitrate reductase (napA) |
| K02568 | cytochrome c-type protein (napB) |
| **Nitrification (K10944+K10945+K10946)/3** | |
| K10944 | ammonia monooxygenase subunit A (amoA) |
| K10945 | ammonia monooxygenase subunit B (amoB) |
| K10946 | ammonia monooxygenase subunit C (amoC) |
| **Nitrogen assimilation (K00360+K00367+K01915+K00265+K00284)/3** | |
| K00265 | glutamate synthase (NADPH/NADH) large chain (gltB) |
| K00284 | glutamate synthase (ferredoxin-dependent) (gltS) |
| K00360 | assimilatory nitrate reductase |
| K00367 | assimilatory nitrate reductase |
| K01915 | glutamine synthetase (glnA) |
| **Nitrogen Fixation (K00531+K02586+K02588+K02591)/4** | |
| K00531 | nitrogenase |
| K02586 | nitrogenase molybdenum-iron protein alpha chain (nifD) |
| K02588 | nitrogenase iron protein (nifH) |
| K02591 | nitrogenase molybdenum-iron protein beta chain (nifK) |
| **Nitrogen Mineralization K00260+K00261+K00262** | |
| K00260 | glutamate dehydrogenase |
| K00261 | glutamate dehydrogenase |
| K00262 | glutamate dehydrogenase |
| **Sulfur Cycle** | |
| **Assimilatory sulfate reduction (K00860+K00956+K00957)/3** | |
| K00860 | adenylylsulfate kinase (cysC) |
| K00956 | sulfate adenylyltransferase subunit 1 (cysN) |
| K00957 | sulfate adenylyltransferase subunit 2 (cysD) |
| **Dissimilatory sulfate reduction and sulfide oxidation (K00394+K00395+K11180)/3** | |
| K00394 | adenylylsulfate reductase subunit A (aprA) |
| K00395 | adenylylsulfate reductase subunit B (aprB) |
| K11180 | sulfite reductase (dsrA) |
| **Sulfur Mineralization K00456+K01011** | |
| K00456 | cysteine dioxygenase |
| K01011 | 3-mercaptopyruvate sulfurtransferase |
| **Polysulfide reduction** | |
| K08352 | polysulfide reductase chain A (psrA) |
| **Phosphorus Cycle** | |
| **Phosphate transport (K02038+K02036+K02037+K02040)/4** | |
| K02038 | phosphate transport system permease protein (pstA) |
| K02036 | phosphate transport system ATP-binding protein (pstB) |
| K02037 | pstC; phosphate transport system permease protein (pstC) |
| K02040 | phosphate transport system substrate-binding protein (pstS) |
| **Phosphonoacetate hydrolase** | |
| K06193 | phosphonoacetate hydrolase (phnA) |
| **2-phosphonopropionate transporter** | |
| K06193 | phosphonoacetate hydrolase (phnA) |
| **Phosphonate transport (K02041+K02044+K02042)/3** | |
| K02041 | phosphonate transport system ATP-binding protein (phnC) |
| K02044 | phosphonate transport system substrate-binding protein (phnD) |
| K02042 | phosphonate transport system permease protein (phnE) |
| **Phosphonate metabolism**  **(K02043+K06166+K06165+K06164+K06163+K05781+K05780+K06162+K05774)/9** | |
| K02043 | GntR family transcriptional regulator, phosphonate transport system regulatory protein (phnF) |
| K06166 | a-D-ribose 1-methylphosphonate 5-triphosphate synthase subunit PhnG (phnG) |
| K06165 | a-D-ribose 1-methylphosphonate 5-triphosphate synthase subunit PhnH (phnH) |
| K06164 | a-D-ribose 1-methylphosphonate 5-triphosphate synthase subunit PhnI (phnI) |
| K06163 | a-D-ribose 1-methylphosphonate 5-phosphate C-P lyase (phnJ) |
| K05781 | phosphonate transport system ATP-binding protein (phnK) |
| K05780 | a-D-ribose 1-methylphosphonate 5-triphosphate synthase subunit PhnL (phnL) |
| K06162 | a-D-ribose 1-methylphosphonate 5-triphosphate diphosphatase (phnM) |
| K05774 | ribose 1,5-bisphosphokinase (phnN) |
| **2-aminoethylphosphonic acid pathway (K03430+K05306)/2** | |
| K03430 | 2-aminoethylphosphonate-pyruvate transaminase (phnW) |
| K05306 | phosphonoacetaldehyde hydrolase (phnX) |
| **Phosphate regulation (K07636+K02039+K07657+K07658)/4** | |
| K07636 | two-component system, OmpR family, phosphate regulon sensor histidine kinase PhoR (phoR) |
| K02039 | phosphate transport system protein (phoU) |
| K07657 | two-component system, OmpR family, phosphate regulon response regulator PhoB (phoB) |
| K07658 | two-component system, OmpR family, alkaline phosphatase synthesis response regulator PhoP (phoB1; phoP) |
| **Alkaline phosphatase (K01077+K01113)/2** | |
| K01077 | alkaline phosphatase (phoA) |
| K01113 | alkaline phosphatase D (phoD) |
| **G3P transporter (K05814+K05813+K05816+K05815)/4** | |
| K05814 | sn-glycerol 3-phosphate transport system permease (ugpA) |
| K05813 | sn-glycerol 3-phosphate transport system substrate-binding (ugpB) |
| K05816 | sn-glycerol 3-phosphate transport system ATP-binding (ugpC) |
| K05815 | sn-glycerol 3-phosphate transport system permease (ugpE) |
| **Glycerophosphodiester phosphodiesterase** | |
| K01126 | glycerophosphoryl diester phosphodiesterase (ugpQ; glpQ) |
| **Polyphosphate kinase** | |
| K00937 | polyphosphate kinase (ppk) |
| **Exopolyphosphatase** | |
| K01524 | exopolyphosphatase / guanosine-5'-triphosphate,3'-diphosphate pyrophosphatase (ppx-gppA) |

Table S2 OTUs with relative abundance above 1% in surface ice (SI) and/or basial ice (BI)

| OTU ID | SI (%) | BI (%) | taxonomy |
| --- | --- | --- | --- |
| OTUs had a relative abundance above 1% in SI | | | |
| OTU5 | 5.50 | 0.43 | p_Cyanobacteria; c_Oxyphotobacteria; o_Pseudanabaenales; f_Pseudanabaenaceae; g_Pseudanabaena_PCC-7429 |
| OTU7 | 4.98 | 0.45 | p_Proteobacteria; c_Gammaproteobacteria; o_Betaproteobacteriales; f_Burkholderiaceae; g_Rhodoferax; s_Ambiguous_taxa |
| OTU6 | 3.59 | 0.18 | p_Cyanobacteria; c_Oxyphotobacteria; o_Leptolyngbyales; f_Leptolyngbyaceae; g_uncultured; s_Ambiguous_taxa |
| OTU14 | 3.11 | 0.54 | p_Bacteroidetes; c_Bacteroidia; o_Cytophagales; f_Hymenobacteraceae; g_Hymenobacter |
| OTU15 | 2.95 | 0.22 | p_Bacteroidetes; c_Bacteroidia; o_Flavobacteriales; f_Flavobacteriaceae; g_Flavobacterium; s_Flavobacterium_sp. |
| OTU791 | 2.88 | 0.38 | p_Proteobacteria; c_Gammaproteobacteria; o_Betaproteobacteriales; f_Burkholderiaceae; g_Polaromonas |
| OTU118 | 2.32 | 0.22 | p_Cyanobacteria; c_Oxyphotobacteria; o_Pseudanabaenales; f_Pseudanabaenaceae; g_Pseudanabaena_PCC-7429 |
| OTU22 | 1.87 | 0.07 | p_Cyanobacteria; c_Oxyphotobacteria; o_Nostocales; f_Phormidiaceae; g_Tychonema_CCAP_1459-11B; s_Ambiguous_taxa |
| OTU8 | 1.75 | 0.12 | p_Bacteroidetes; c_Bacteroidia; o_Chitinophagales; f_Chitinophagaceae; g_Ferruginibacter |
| OTU31 | 1.47 | 0.08 | p_Proteobacteria; c_Gammaproteobacteria; o_Betaproteobacteriales; f_Methylophilaceae; g_Methylotenera; s_uncultured_bacterium |
| OTU789 | 1.43 | 0.23 | p_Bacteroidetes; c_Bacteroidia; o_Cytophagales; f_Hymenobacteraceae; g_Hymenobacter |
| OTU29 | 1.20 | 0.16 | p_Bacteroidetes; c_Bacteroidia; o_Cytophagales; f_Spirosomaceae; g_Arcicella; s_uncultured_bacterium |
| OTU36 | 1.08 | 0.03 | p_Firmicutes; c_Clostridia; o_Clostridiales; f_Ruminococcaceae; g_Intestinimonas; s_uncultured_bacterium |
| OTU20 | 1.02 | 0.10 | p_Cyanobacteria; c_Oxyphotobacteria; o_Leptolyngbyales; f_Leptolyngbyaceae; g_Chamaesiphon_PCC-7430; s_Ambiguous_taxa |
| OTU25 | 1.02 | 0.11 | p_Proteobacteria; c_Alphaproteobacteria; o_Sphingomonadales; f_Sphingomonadaceae; g_Sphingorhabdus; s_Ambiguous_taxa |
| OTU27 | 1.01 | 0.17 | p_Proteobacteria; c_Gammaproteobacteria; o_Betaproteobacteriales; f_Burkholderiaceae; g_Polaromonas |
| OTUs had a relative abundance above 1% in BI | | | |
| OTU18 | 0.06 | 3.24 | p_Firmicutes; c_Bacilli; o_Lactobacillales; f_Streptococcaceae; g_Streptococcus |
| OTU16 | 0.68 | 2.85 | p_Firmicutes; c_Bacilli; o_Lactobacillales; f_Streptococcaceae; g_Lactococcus; s_Lactococcus_garvieae_subsp._garvieae |
| OTU56 | 0.21 | 2.51 | p_Firmicutes; c_Bacilli; o_Lactobacillales; f_Enterococcaceae; g_Enterococcus |
| OTU94 | 0.47 | 2.44 | p_Proteobacteria; c_Gammaproteobacteria; o_Enterobacteriales; f_Enterobacteriaceae |
| OTU47 | 0.10 | 1.75 | p_Proteobacteria; c_Gammaproteobacteria; o_Enterobacteriales; f_Enterobacteriaceae; g_Klebsiella |
| OTU185 | 0.08 | 1.74 | p_Firmicutes; c_Bacilli; o_Bacillales; f_Bacillaceae; g_Bacillus |
| OTU105 | 0.19 | 1.71 | p_Proteobacteria; c_Gammaproteobacteria; o_Enterobacteriales; f_Enterobacteriaceae; g_Pantoea |
| OTU33 | 0.20 | 1.46 | p_Firmicutes; c_Bacilli; o_Bacillales; f_Bacillaceae; g_Bacillus |
| OTU70 | 0.38 | 1.29 | p_Firmicutes; c_Bacilli; o_Lactobacillales; f_Lactobacillaceae; g_Lactobacillus; s_Lactobacillus_gasseri |
| OTU837 | 0.21 | 1.10 | p_Proteobacteria; c_Gammaproteobacteria; o_Enterobacteriales; f_Enterobacteriaceae |
| OTU188 | 0.02 | 1.08 | p_Proteobacteria; c_Gammaproteobacteria; o_Enterobacteriales; f_Enterobacteriaceae; g_Klebsiella |
| OTUs had a relative abundance above 1% in both SI and BI | | | |
| OTU1 | 4.25 | 23.99 | p_Firmicutes; c_Bacilli; o_Lactobacillales; f_Lactobacillaceae; g_Lactobacillus |
| OTU10 | 1.20 | 4.06 | p_Firmicutes; c_Bacilli; o_Lactobacillales; f_Streptococcaceae; g_Lactococcus; s_Lactococcus_lactis |
| OTU17 | 1.65 | 1.17 | p_Firmicutes; c_Bacilli; o_Lactobacillales; f_Leuconostocaceae; g_Weissella |
| OTU2 | 12.19 | 1.01 | p_Proteobacteria; c_Gammaproteobacteria; o_Betaproteobacteriales; f_Burkholderiaceae; g_Polaromonas; s_uncultured_bacterium |
| OTU3 | 1.85 | 6.87 | p_Firmicutes; c_Bacilli; o_Lactobacillales; f_Lactobacillaceae; g_Lactobacillus; s_Lactobacillus_brevis |
| OTU4 | 3.65 | 9.18 | p_Firmicutes; c_Bacilli; o_Lactobacillales; f_Lactobacillaceae; g_Pediococcus; s_Pediococcus_pentosaceus |
| OTU48 | 1.41 | 1.63 | p_Firmicutes; c_Bacilli; o_Lactobacillales; f_Lactobacillaceae; g_Lactobacillus; s_Lactobacillus_aviarius |
| OTU9 | 1.27 | 5.43 | p_Firmicutes; c_Bacilli; o_Bacillales; f_Bacillaceae; g_Bacillus |
